# T-Regulatory Cells in Health and Disease

**DOI:** 10.1155/2018/5025238

**Published:** 2018-08-05

**Authors:** Eyad Elkord, Varun Sasidharan Nair

**Affiliations:** Cancer Research Center, Qatar Biomedical Research Institute, College of Science and Engineering, Hamad Bin Khalifa University, Qatar Foundation, Doha, Qatar

Different mechanisms of the immune system are pivotal to discriminate self-antigens from non-self antigens in order to maintain tolerance and induce protective immunity against foreign antigens. T regulatory cells (Tregs) are essential for peripheral tolerance but they contribute to the immunopathogenesis of different diseases including autoimmunity, allergy, inflammation, graft rejection, and cancer.

We are pleased to introduce this special issue, bringing 11 contributions from different research groups. These articles advance our understanding on the role and function of Tregs in different pathological conditions.

Several studies reported that Tregs are induced and/or expanded in the tumor microenvironment, where they can suppress antitumor immune responses and contribute to tumor progression and poor prognosis [1–4]. In this issue, an article by M. Niedźwiecki et al. reported a statistically higher level of FoxP3^+^ Tregs in the bone marrow than in peripheral blood of a group of 42 children with acute lymphoblastic leukemia, which might be favorable for the development of leukemic bone marrow at early stages. This higher level of Tregs in bone marrow could be a potential risk factor for poor prognosis of hematological malignancies. In another article addressing the importance of Tregs in hematological malignancies, M. Delia et al. investigated the percentage of Tregs in the diagnostic bone marrow aspirates (dBMA) and their correlation with response to chemotherapy and survival in 23 acute myeloid leukemia (AML) patients. They reported that higher Treg numbers in dBMA predicted better response and survival of AML patients. This study suggests a prognostic role for Tregs in AML patients receiving intensive chemotherapy. In the following review article, N. Hosaka discussed the role of Tregs in tumor-bearing mice treated with allo-hematopoietic stem cell transplantation plus thymus transplantation. This review highlights the importance of Tregs in the enhanced graft-versus-tumor effect and reduction of graft-versus-host disease, leading to a better outcome and longer survival.

Forkhead box P3 (FoxP3) is the master transcription factor for Treg development and function [5]. Mutations in FoxP3 lead to substantial decrease in Tregs, which results in severe autoimmune disorders [6]. In this special issue, five articles give us more insights into the importance of Tregs in autoimmune disorders. H. Keino et al. present an interesting and comprehensive review on the ocular immune privilege associated with eye-derived Tregs. The authors provided a robust background on the molecular mechanisms responsible for the development and maintenance of ocular immune privilege by Tregs. Clearly, further understanding of the ocular immune privilege associated with Tregs could offer a new approach to therapeutic interventions for ocular autoimmunity. Next review article by D. Calzada et al. reviewed different mechanisms of Tregs involved in allergy and allergen tolerance. The authors provided an update on the function of Tregs in allergic diseases and the potential use of Tregs as novel therapeutic approaches. The next review article by D. Vdovenko and U. Eriksson focused on the role of Tregs in myocarditis. The authors concluded that different CD4^+^ T effector cells including Th1 and Th17 have a critical role in myocarditis, and Tregs have a specific role to limit disease progression. However, understanding the specific roles of T cell subpopulations at different stages of the disease progression is critical for the development of successful therapeutic strategies. Next, T. Chen et al. showed that in the peripheral blood of asthmatic patients, apart from CD25^+^FOXP3^+^ canonical Tregs, there is a distinct population of Tregs which express a higher level of GATA3. Additionally, authors showed that in patients, both the percentage and immunosuppressive function of canonical Tregs were highly impaired due to the elevated expression of these Th2-like Tregs. However, the role of GATA3^+^/FOXP3^+^ Th2-like Tregs is still not fully disclosed. In another article, M. Vitales-Noyola et al. assessed the influence of low versus high sodium intake on different immunological parameters, especially on Tregs and Th17 subsets, in patients with rheumatic arthritis (RA) and systemic lupus erythematosus (SLE). The authors concluded that the level of sodium intake is not associated with different immune parameters in healthy donors or patients with SLE or RA.

The last group of these articles is categorized under transplantation biology. The role of Tregs in transplantation is to create tolerance and suppress graft-versus-host disease. N. Pilat et al. described the effect of CTLA4Ig on induction and suppressive function of mouse-induced Tregs (iTregs) in *in vitro* culture. CTLA4Ig has been approved for the treatment of autoimmune diseases and transplant rejection. The authors reported that the costimulation blocker significantly improves the generation and suppressive function of iTregs. Moreover, these iTregs could be a better choice for graft survival over calcineurin-based immunosuppressive regimens. Next, M. Vieyra-Lobato et al. comprehensively described the role of CD8^+^ Tregs in various disease aspects. This review discussed different surface markers including Treg signatures and cytokines in several diseases including autoimmunity, cancer, and graft-versus-host disease as a strategy in their prevention, monitoring, and cure. In another article, T. E. C. Kieffer et al. showed that pregnancies with male fetuses more often lead to pregnancy complications such as preterm birth and preeclampsia. The authors suggested that in male fetuses, there exist a Y chromosome-associated pathophysiology consisting of lower FOXP3, IFN-*γ*, and IL-6 mRNA expression, leading to a high complication risk during pregnancy, which was absent in females. This study advances our current knowledge in reproductive immunology regarding the immunologic differences between male and female fetuses with a divergent pathophysiology outcome.

In summary, a considerable progress has been made to understand both genetic and phenotypic changes acquired in Tregs at different disease aspects. We hope that the articles in this special issue improve our understanding on the role and function of different Treg subsets in various disease settings. Further underlying mechanisms behind the phenotypic and functional changes acquired by Tregs in various diseases should be revealed, which could provide insights to target them and enhance different therapeutic strategies.
